# Impact of Coronary Dominance on In-Hospital Outcomes after Percutaneous Coronary Intervention in Patients with Acute Coronary Syndrome

**DOI:** 10.1371/journal.pone.0072672

**Published:** 2013-08-26

**Authors:** Toshiki Kuno, Yohei Numasawa, Hiroaki Miyata, Toshiyuki Takahashi, Koichiro Sueyoshi, Takahiro Ohki, Koji Negishi, Akio Kawamura, Shun Kohsaka, Keiichi Fukuda

**Affiliations:** 1 Department of Cardiology, Ashikaga Red Cross Hospital, Tochigi, Japan; 2 University of Tokyo, Healthcare Quality Assessment, Tokyo, Japan; 3 Department of Cardiology, Kawasaki City Municipal Hospital, Kanagawa, Japan; 4 Department of Cardiology, Tokyo Dental College Ichikawa General Hospital, Chiba, Japan; 5 Department of Cardiology, Yokohama Municipal Hospital, Kanagawa, Japan; 6 Department of Cardiology, Keio University School of Medicine, Tokyo, Japan; University Medical Center Utrecht, Netherlands

## Abstract

**Objective:**

This study evaluated the manner in which coronary dominance affects in-hospital outcomes of acute coronary syndrome (ACS) patients undergoing percutaneous coronary intervention (PCI).

**Background:**

Previous studies have shown that left dominant coronary anatomies are associated with worse prognoses in patients with coronary artery disease.

**Methods:**

Data were analyzed from 4873 ACS patients undergoing PCI between September 2008 and April 2013 at 14 hospitals participating in the Japanese Cardiovascular Database Registry. The patients were grouped based on diagnostic coronary angiograms performed prior to PCI; those with right- or co-dominant anatomy (RD group) and those with left-dominant anatomy (LD group).

**Results:**

The average patient age was 67.6±11.8 years and both patient groups had similar ages, coronary risk factors, comorbidities, and prior histories. The numbers of patients presenting with symptoms of heart failure, cardiogenic shock, or cardiopulmonary arrest were significantly higher in the LD group than in the RD group (heart failure: 650 RD patients [14.7%] vs. 87 LD patients [18.8%], *P* = 0.025; cardiogenic shock: 322 RD patients [7.3%] vs. 48 LD patients [10.3%], *P* = 0.021; and cardiopulmonary arrest: 197 RD patients [4.5%] vs. 36 LD patients [7.8%], *P* = 0.003). In-hospital mortality was significantly higher among LD patients than among RD patients (182 RD patients [4.1%] vs. 36 LD patients [7.8%], *P* = 0.001). Multivariate logistic regression analysis revealed that LD anatomy was an independent predictor for in-hospital mortality (odds ratio, 1.75; 95% confidence interval, 1.06–2.89; *P = *0.030).

**Conclusion:**

Among ACS patients who underwent PCI, LD patients had significantly worse in-hospital outcomes compared with RD patients, and LD anatomy was an independent predictor of in-hospital mortality.

## Introduction

Variations in the balance of the coronary arteries are common, particularly with regard to the supply of the posterior aspect of the left ventricle. In the majority of patients, the right coronary artery (RCA) reaches the crux of the heart and supplies the posterior descending artery (PDA) [1]. Left-dominant (LD) anatomy, described as a variant of normal anatomy, has a prevalence of approximately 5–12% in the general population [2]–[5]. In these individuals, the left circumflex artery (LCX) reaches the crux and supplies the posterior descending and, usually, the atrioventricular nodal branches [2].

LD anatomy is believed to be associated with worse prognoses for patients with acute coronary syndrome (ACS) and stable coronary artery disease [3], [6]. The PDA, arising from the RCA, may serve as a back-up supply in normal anatomy, with the right-dominant (RD) system acting in an overall protective manner. LD patients, in contrast, usually have only the left coronary artery to supply the majority of the myocardium. Thus, an event in a major vessel may, therefore, lead to a worse outcome. Furthermore, technical challenges exist in interventions associated with the LCX artery because of the steepness of its take-off, relative to other arteries [7]. However, data describing the effects of coronary dominance in modern percutaneous coronary intervention (PCI) are scarce.

## Methods

### Study Design

The Japan Cardiovascular Database (JCD) is a large, ongoing, prospective, multicenter, cohort study designed to collect clinical background and outcome data on PCI patients. Data pertaining to approximately 150 variables are being collected. In this registry, participating hospitals have been instructed to record data from hospital visits for consecutive PCI patients and to register these data into an internet-based database. The database system performs checks to ensure that the reported data are complete and internally consistent. PCIs performed using any commercially available coronary device may be included. The decision to perform PCI is made according to the attending physicians’ clinical assessments of the patient. The study does not mandate specific interventional or surgical techniques, such as vascular access, or the use of a specific stent or closure device.

The majority of the clinical variables in the JCD were defined according to the National Cardiovascular Data Registry (NCDR), sponsored by the American College of Cardiology, to conduct comparative research and determine the factors that lead to disparities in PCI management. NCDR is a large PCI registry system with over 1,000,000 entries of ischemic heart disease and over 500,000 PCI entries collected from more than 500 institutions in the USA [8], [9].

Cardiogenic shock was defined as a sustained (>30 minutes) episode of systolic blood pressure <90 mm Hg, and/or a cardiac index of <2.2 L/min/m^2^ determined to be secondary to cardiac dysfunction, and/or the requirement for parenteral inotropic or vasopressor agents or mechanical support (e.g., intra-aortic balloon pump [IABP], extracorporeal circulation, ventricular assist devices) to maintain blood pressure and a cardiac index above the levels specified. Heart failure was defined as physician documentation or reported clinical symptoms of heart failure, such as unusual dyspnea on light exertion, recurrent dyspnea occurring in the supine position, fluid retention; or the description of rales, jugular venous distension, or pulmonary edema on physical examination; or pulmonary edema evident in chest radiographs and presumed to be associated with cardiac dysfunction. A low ejection fraction, without clinical evidence of heart failure, did not qualify as heart failure. Cardiopulmonary arrest was defined as cardiac arrest and/or respiratory arrest before the PCI procedure.

PCI for urgent therapy was defined as a procedure performed on an inpatient basis because of significant concerns regarding a risk of ischemia, infarction, and/or death. However, urgent PCI was also performed on outpatients or emergency department patients when a cardiac catheterization was required and warranted an admission, based on the clinical presentation. PCI for emergent therapy was defined as a procedure that is performed as soon as possible because of substantial concerns that ongoing ischemia and/or infarction could lead to death. The phrase “as soon as possible” referred to a situation wherein a patient has a clinical presentation of sufficient gravity that a scheduled case would be canceled to perform this procedure. PCI for salvage therapy was defined as a procedure that was performed as a last resort; in such cases, the patient was in cardiogenic shock at the start of the procedure or within the 10 minutes prior to the start of the procedure, and the patient had also received chest compressions for a total of at least 60 s or had been on unanticipated extracorporeal circulatory support (e.g., extracorporeal membrane oxygenation, cardiopulmonary support).

### Information Disclosure

Before the launch of the JCD, information on the objectives of the study, its social significance, and an abstract were provided to register this clinical trial with the University Hospital Medical Information Network. This Network is recognized by the International Committee of Medical Journal Editors as an “acceptable registry,” according to a statement issued in September 2004 (UMIN R000005598).

### JCD Participants

Major teaching hospitals within the metropolitan Tokyo area were selected for the pilot phase of this study, and the study protocol was approved by the institutional review board (IRB) committee at each site. In this registry, the data were collected since September 2008 from the 14 Japanese hospitals participating in the Japanese Cardiovascular Database (JCD) [10]–[13]. All patients aged >18 years undergoing PCI or an acetylcholine challenge test in these hospitals were enrolled.

### Procedures and Data Collection

In this study, all ACS patients who underwent PCI were included. We did not include those who underwent acetylcholine challenge tests in our analysis since, typically, patients undergo this challenge test only if they do not have obstructive lesions and vasospastic angina is suspected [14], [15].

Data were analyzed from the 4873 patients undergoing PCI for ACS at one of the 14 Japanese hospitals participating in the JCD-Keio Interhospital Cardiology Study (KICS) between September 2008 and April 2013.

ACS is used to describe ST-elevation myocardial infarction (STEMI), non ST-elevation myocardial infarction (NSTEMI), and unstable angina (UA). STEMI was defined as myocardial infarction with ST elevation. These elements are equivalent to the definitions provided in the associated guidelines [8], [9], [16]. When anterior lead-ST depression was documented as ‘STEMI equivalent’ by the attending cardiologist, they were also coded as STEMI. On the other hand, if there was no written documentation of STEMI in cases of V1–3 ST depression, those cases were coded NSTEMI.

Patients included in the study were divided into 2 groups, based on a diagnostic coronary angiogram performed prior to their PCI; patients with right- or co-dominant anatomies were placed into the RD group and those with left-dominant anatomies were included in the LD group. RD and co-dominant patients were placed into the same group because these patients have been demonstrated to have similar prognoses [3]. Moreover, the hypothesis that PDAs, arising from the right coronary artery, may serve as a back-up blood supply in individuals with normal anatomy, would indicate that individuals with a co-dominant anatomy would be expected to derive similar benefits to those with RD anatomy. LD anatomy was defined as one in which the PDA originated from the LCX. RD anatomy was defined as the PDA originating from the RCA. Co-dominant anatomy was defined when only the PDA originated from the RCA and the RCA did not give rise to posterolateral branches, in combination with a large posterolateral branch originating from the LCX reaching near the posterior interventricular groove [6]. The dominance of each patient was documented by the treating cardiologist and failure to document dominance information was detected by the clinical coordinator and its input was mandated by the site data manager.

This research was supported by a grant from the Ministry of Education, Culture, Sports, Science, and Technology, Japan (KAKENHI No. 21790751). The JCD Steering Committee was responsible for overall study guidance, including the study protocol, data analyses, and interpretation of results. The Department of Healthcare Quality Assessment at Tokyo University independently managed the database. The KICS Committee managed the participating sites and provided monthly on-site monitoring services to assure data accuracy and completeness throughout the study. During the planning, implementation, and reporting of this study, there were no issues such as conflicts of interest, conflicts of responsibility, or intellectual property right concerns.

The study endpoints included in-hospital mortality, heart failure, cardiogenic shock, and other complications. Complications were defined as all complications, including severe dissection or coronary perforation, myocardial infarction after PCI, contrast-induced nephritis, cardiogenic shock or heart failure, cerebral bleeding or stroke, and bleeding complications. Bleeding complications in this registry were defined as those requiring transfusion, prolonging hospital stay, and/or causing a decrease in hemoglobin of >3.0 g/dL [17]. Further, bleeding complications were subdivided into puncture-site bleeding, retroperitoneal bleeding, gastrointestinal bleeding, genitourinary bleeding, or other bleeding.

### Data Analyses

Continuous variables are expressed as means and standard deviations (SD); categorical variables are expressed as percentages. Continuous variables were compared using Student’s *t*-test, and differences between categorical variables were examined using a chi-squared test. A multiple logistic regression analysis was performed to determine the independent predictors of in-hospital mortality. Factors, at admission, that were evaluated in the multivariate model were age, 70–79 years; age, ≥80 years; body mass index (BMI) ≥ 30; presence of chronic kidney disease stage ≥ 3; presence of chronic obstructive pulmonary disease; presence of Canadian Cardiovascular Society class 4 angina; dialysis dependence; presence of heart failure; cardiogenic shock; cardiopulmonary arrest; left main trunk stenosis; STEMI; NSTEMI; PCI for emergent therapy; PCI for salvage therapy; and LD anatomy. All statistical calculations and analyses were performed using SPSS version 15 (SPSS, Chicago, IL, USA). *P*-values of <0.05 were considered statistically significant.

## Results

A total of 4873 ACS patients undergoing PCI were assessed. The average age of the patients was 67.6±11.8 years and 1081 (22.2%) patients were female. The numbers of STEMI, NSTEMI, and UA patients were 2334 (47.9%), 778 (16.0%), and 1761 (36.1%), respectively. The LD group was comprised of 464 patients (9.5%), and there were 4409 patients (90.5%) in the RD group. Among 4873 ACS patients, 639 (12.4%) had co-dominant anatomy.

### Baseline Characteristics

The baseline clinical characteristics of the 4873 patients in both groups are summarized in Table 1. There were no major differences in the coronary risk factors, BMI, comorbidities (cerebrovascular disease, chronic obstructive pulmonary disease, presence of chronic kidney disease stage ≥3, hemodialysis, peripheral artery disease), or past history (prior myocardial infarction, prior heart failure, prior PCI, prior coronary artery bypass grafting). The numbers of patients presenting with symptoms of heart failure, cardiogenic shock, or cardiopulmonary arrest were significantly higher in the LD group than in the RD group (heart failure: 650 RD patients [14.7%] vs. 87 LD patients [18.8%], *P* = 0.025; cardiogenic shock: 322 RD patients [7.3%] vs. 48 LD patients [10.3%], *P* = 0.021; and cardiopulmonary arrest: 197 RD patients [4.5%] vs. 36 LD patients [7.8%], *P* = 0.003) (Table 1).

**Table 1:** Baseline clinical characteristics of each group.

	RD+Co %(n = 4409)	LD %(n = 464)	*P* Value
Age ≥80 years	16.2 (714)	18.3 (85)	0.236
Age 70–79 years	32.3 (1422)	32.8 (152)	0.835
Female	22.4 (989)	19.8 (92)	0.217
BMI ≥30	5.4 (240)	4.7 (22)	0.589
Coronary risk factors			
DM	37.2 (1642)	37.7 (175)	0.840
DM with insulin	6.7 (297)	8.4 (39)	0.178
Hypertension	70.1 (3089)	69.0 (320)	0.632
Hyperlipidemia	61.2 (2699)	58.6 (272)	0.293
Smoking	39.2 (1730)	41.8 (194)	0.294
Comorbidities			
CVD	8.5 (375)	9.3 (43)	0.601
COPD	2.7 (120)	4.3 (20)	0.057
CKD stage ≥ 3	17.5 (770)	18.5 (86)	0.564
Hemodialysis	3.8 (166)	2.8 (13)	0.363
PAD	5.6 (248)	6.3 (29)	0.598
History			
Prior MI	16.3 (718)	15.1 (70)	0.551
Prior HF	6.5 (286)	7.1 (33)	0.621
Prior PCI	20.6 (910)	19.6 (91)	0.629
Prior CABG	4.1 (179)	2.4 (11)	0.077
Presenting status			
CCS class 4 angina	15.4 (681)	16.6 (77)	0.501
HF	14.7 (650)	18.8 (87)	0.025
NYHA class 3/4	9.7 (426)	15.3 (71)	<0.001
Cardiogenic shock	7.3 (322)	10.3 (48)	0.021
CPA	4.5 (197)	7.8 (36)	0.003

RD, right dominant; Co, co-dominant; LD, left dominant; BMI, body mass index; DM, diabetes mellitus; CVD, cerebrovascular disease; COPD, chronic obstructive pulmonary disease; CKD, chronic kidney disease; PAD, peripheral artery disease; MI, myocardial infarction; HF, heart failure; PCI, percutaneous coronary intervention; CABG, coronary artery bypass grafting; CCS, Canadian Cardiovascular Society; NYHA, New York Heart Association; CPA, cardiac pulmonary arrest.

### Angiographical Data and Procedural Information

The angiographical and procedural data are listed in Table 2. The number of patients with STEMI, NSTEMI, and UA were similar between the groups. There were no statistically significant differences in the proportions of drug eluting stents implanted (2387 RD patients [54.1%] vs. 259 LD patients [55.8%], *P* = 0.493), nor in the proportions of bare metal stents implanted (1493 RD patients [33.9%] vs. 143 LD patients [30.8%], *P* = 0.197).

**Table 2:** Procedural information for each group.

	RD+Co %(n = 4409)	LD %(n = 464)	*P* Value
Coronary status			
STEMI	48.2 (2125)	45.0 (209)	0.204
NSTEMI	15.7 (692)	18.5 (86)	0.125
UA	36.1 (1592)	36.4 (169)	0.919
2-Vessel disease	42.9 (1890)	46.1 (214)	0.184
3-Vessel disease	24.7 (1088)	22.2 (103)	0.256
LMT stenosis	9.3 (410)	6.9 (32)	0.089
PCI indication			
Emergent therapy	48.3 (2129)	49.1 (228)	0.733
Salvage therapy	3.5 (154)	6.7 (31)	0.002
Stent Implantation			
DES	54.1 (2387)	55.8 (259)	0.493
BMS	33.9 (1493)	30.8 (143)	0.197
Balloon angioplasty	7.1 (314)	8.4 (39)	0.301
Puncture site			
Radial artery	23.9 (1053)	22.0 (102)	0.389
Femoral artery	74.2 (3273)	76.7 (356)	0.263
IABP insertion			
Before PCI	2.9 (126)	2.8 (13)	0.945
During/after PCI	10.2 (449)	13.8 (64)	0.016

RD, right dominant; Co, co-dominant; LD, left dominant; STEMI, ST elevation myocardial infarction; NSTEMI, non-ST elevation myocardial infarction; UA, unstable angina; LMT, left main trunk; PCI, percutaneous coronary intervention; DES, drug eluting stent; BMS, bare metal stent; IABP, intra-aortic balloon pump.

The proportions of IABPs used were analyzed in both groups. The numbers of IABP insertions before PCI procedures were similar in both groups (126 RD patients [2.9%] vs. 13 LD patients [2.8%], *P* = 0.945). However, the number of IABP insertions during and after PCI procedures was significantly higher in the LD group than in the RD group (449 RD patients [10.2%] vs. 64 LD patients [13.8%], *P* = 0.016).

### In-hospital Outcomes/Complications

Table 3 shows the overall in-hospital outcomes and complications for the 2 groups of patients. The in-hospital mortality rate was significantly higher in the LD group compared to the RD group (182 RD patients [4.1%] vs. 36 LD patients [7.8%], *P* = 0.001) (Table 3). The combined rates of heart failure, cardiogenic shock, and death were also significantly higher in the LD group than in the RD group (349 RD patients [7.9%] vs. 53 LD patients [11.4%], *P* = 0.013). As listed in Table 3, the rates of thrombolysis in myocardial infarction (TIMI) flow that were<grade 3, the rates of hemoglobin decreases of >3 g/dL, the rates of contrast-induced nephritis, and the rates of other complications, including bleeding complications, were similar for the 2 groups. Only gastrointestinal bleeding complications were significantly different between the groups (28 RD patients [0.6%] vs. 7 LD patients [1.5%], *P* = 0.044).

**Table 3:** In-hospital outcome/complications in each group.

	RD+Co % (n = 4409)	LD % (n = 464)	*P* Value
All-cause death	4.1 (182)	7.8 (36)	0.001
HF/shock/death	7.9 (349)	11.4 (53)	0.013
TIMI flow grade <3	6.2 (273)	6.7 (31)	0.686
HB, >3 g/dL decrease	14.0 (555)	13.7 (57)	0.941
CIN	19.6 (864)	87 (18.8)	0.712
Any complications	13.6 (600)	15.1 (70)	0.395
Dissection	1.5 (52)	1.1 (5)	1.000
Perforation	0.7 (33)	0.9 (4)	0.776
MI	2.1 (92)	2.4 (11)	0.613
HF	3.3 (146)	4.5 (21)	0.179
Shock	3.2 (142)	3.9 (18)	0.413
Dialysis	1.8 (79)	0.9 (4)	0.184
Stroke	0.7 (31)	0.4 (2)	0.765
ICH	0.1 (4)	0.0 (0)	1.000
Transfusion	3.7 (161)	4.3 (20)	0.440
Bleeding	4.1 (179)	5.8 (27)	0.088
Puncture site bleeding	1.4 (61)	2.4 (11)	0.103
Hematoma	1.0 (43)	0.6 (3)	0.620
Retroperitoneal	0.1 (4)	0.2 (1)	0.394
Gastrointestinal	0.6 (28)	1.5 (7)	0.044
Genitourinary	0.3 (12)	0.6 (3)	0.166
Other	1.5 (68)	1.7 (8)	0.694

RD, right dominant; Co, co-dominant; LD, left dominant; TIMI, thrombolysis in myocardial infarction; HF, heart failure; Hb, hemoglobin; CIN, contrast induced nephritis; MI, myocardial infarction; ICH, intracranial hemorrhage.

Multivariate logistic regression analysis showed that LD anatomy was one of the independent predictors for in-hospital mortality (odds ratio, 1.75; 95% confidence interval, 1.06–2.89; *P = *0.030). Cardiogenic shock, heart failure, and cardiopulmonary arrest were also independent predictors for in-hospital mortality (Table 4).

**Table 4:** Multivariate logistic regression analysis on in-hospital mortality.

	OR	95% CI	*P* Value
Left dominance	1.75	1.06–2.89	0.030
Age, ≥80	3.64	2.40–5.53	<0.001
Age 70–79	1.61	1.08–2.41	0.02
BMI ≥30	2.23	1.20–4.46	0.023
CKD stage ≥3	3.48	2.33–5.20	<0.001
CCS class 4 angina	2.11	1.12–3.99	0.021
STEMI	4.59	2.16–9.74	<0.001
NSTEMI	3.58	1.68–7.65	0.001
Cardiogenic shock	2.79	1.69–4.61	<0.001
HF	2.54	1.73–3.71	<0.001
CPA	2.42	1.21–4.85	0.012
LMT stenosis	1.85	1.15–2.97	0.011

OR, odds ratio; CI, confidence interval; BMI, body mass index; CKD, chronic kidney disease; CCS, Canadian Cardiovascular Society; STEMI, ST elevation myocardial infarction; NSTEMI, non-ST elevation myocardial infarction; HF, heart failure; CPA, cardiac pulmonary arrest; LMT, left main trunk.

### In-hospital Outcomes in Selected Subgroups

Tables 5 and 6 show the results from the data analysis conducted in selected subgroups of high-risk patients. LD patients tended to have higher rates of in-hospital mortality for patients with cardiogenic shock and/or cardiopulmonary arrest (101 RD patients [27.2%] vs. 21 LD patients [38.9%], *P* = 0.106) (Table 5), or left main trunk stenosis (44 RD patients [10.7%] vs. 6 LD patients [18.8%], *P* = 0.239) (Table 6).

**Table 5:** In-hospital outcomes/complications in patients with cardiogenic shock and/or cardiopulmonary arrest.

	RD+Co % (n = 371)	LD % (n = 54)	*P* Value
All-cause death	27.2 (101)	38.9 (21)	0.106
HF/shock/death	36.1 (134)	46.3 (25)	0.176
TIMI flow grade <3	14.6 (54)	13.0 (7)	1.000
Bleeding	12.1 (45)	24.1 (13)	0.031
CIN	31.8 (118)	33.3 (18)	0.876

RD, right dominant; Co, co-dominant; LD, left dominant; HF, heart failure; TIMI, thrombolysis in myocardial infarction; Hb, hemoglobin; CIN, contrast induced nephritis.

**Table 6:** In-hospital outcomes/complications in patients with left main trunk stenosis.

	RD+Co % (n = 410)	LD % (n = 32)	*P* Value
All-cause death	10.7 (44)	18.8 (6)	0.239
HF/shock/death	17.8 (73)	18.8 (6)	0.814
TIMI flow grade <3	9.0 (37)	12.5 (4)	0.523
Bleeding	7.8 (32)	3.1 (1)	0.496
CIN	26.1 (107)	15.6 (5)	0.213

RD, right dominant; Co, co-dominant; LD, left dominant; HF, heart failure; TIMI, thrombolysis in myocardial infarction; Hb, hemoglobin; CIN, contrast induced nephritis.

## Discussion

The present study concluded that coronary dominance affected in-hospital outcomes. LD patients had significantly worse in-hospital outcomes compared with RD patients and LD anatomy was an independent predictor of in-hospital mortality in the multivariate analysis. The significance of coronary dominance should be taken into consideration when treating ACS patients with PCI. The absence of the protective effects of a double supply to the myocardium and technical challenges caused by the anatomy may be particularly important in these patients.

Previous studies also have shown that LD patients with coronary artery disease may have worse prognoses than RD patients with coronary artery disease. Goldberg et al. demonstrated that LD is a significant and independent predictor of increased long-term mortality in patients with ACS [3]. However, in their study, the revascularization rate (by PCI or coronary artery bypass graft [CABG]) was approximately 60% and the number of patients with stent implantation was not reported. In the present study, PCI was attempted in all patients.

The higher in-hospital mortality associated with LD patients led to the hypothesis that the RCA serves as a back-up supply in patients with RD anatomy, providing a measure of protection for the myocardium in ACS patients. According to one study [18], patients with acute occlusion of the LCX, presenting with NSTEMI, had better outcomes than did those with STEMI. The analysis indicated that patients with RD anatomies were more likely to have NSTEMI. In concordance with our study, the authors asserted that RD may confer a protective effect in cases of acute occlusion of the LCX, minimizing the infarct size. The data also revealed that smaller infarct sizes and higher likelihoods of RD were associated with NSTEMI patients, supporting their conclusion.

In general, a dominant LCX has several acute angles in its course, including at its origin and at its distal end where it becomes the PDA. These acute angles lead to turbulence and shear stress during blood flow that, in turn, may enhance thrombus formation and platelet activity [19], [20]. The acute angles, and resultant turbulence and shear stress, also contribute to the difficulty of LCX interventions. Yip et al. concluded that LD is related to unsuccessful reperfusion and to a higher 30-day mortality rate in LCX infarct-related acute myocardial infarction (MI) patients [21]. In addition, Auriti et al. showed that coronary flow reserve was more impaired in LCX than LAD just after Y-graft intervention, which was a graft formed by the left internal mammary artery (LIMA) connected to the LAD and by a free right internal mammary artery (RIMA) connected to LIMA and a marginal artery of the LCX [22]. Clinically, a recent study showed that the 30-day prognostic outcome was less favorable in LCX-related acute inferior MI compared to RCA-related acute inferior MI [23]. The reasons for this difference were suggested to include higher peak levels of creatine kinase-MB isoenzyme, lower left ventricular ejection fractions, and the higher numbers of advanced congestive heart failure that were observed in LCX-related acute inferior MIs. Lower rates of stent implantation and collateral circulation might also contribute to the worse outcomes. The study data did not indicate the manner in which dominance might affect mortality; Kim concluded patients with an occluded LCX presented with less ST elevation and primary PCI [24]. Multivariate analysis showed that primary PCI decreased the hospital mortality for patients with occluded coronary arteries. For these reasons, we suspect that more flow disturbances are induced, leading to more adverse effects on hemodynamics during PCI, in patients with PCI of a dominant LCX.

Larger infarct size is another reason affecting in-hospital mortality for LD patients. IABPs might be effective for limiting infarct sizes in such cases. Pierrakos et al. showed that during reperfusion, IABPs increased coronary blood flow, and effectively limited infarct size [25]. Our study showed that the proportion of IABP insertions during and after PCI procedures was significantly higher in the LD group than in the RD group. We suspect that the LD group tended to have hemodynamic instability more frequently than the RD group during the procedure because of the single coronary supply. Moreover, to overcome the difficulty for intervening LD LCX arteries with PCI, CABG would be considered. However, further prospective study will be necessary.

### Study Limitations

Several limitations may exist in this study. First, this study was an analysis of a multicenter cohort study rather than an observational, non-randomized trial. Second, the study population was of limited size, compared to the number of sites included in our registry. Third, we did not analyze whether the target vessel lesions compromised PDA function. Lastly, all candidates underwent PCI procedures. Some patients with critical conditions, such as cardiogenic shock or left main trunk stenosis, are more favorable candidates for CABG than for PCI and these patients, who underwent CABG instead of PCI, were excluded. Therefore, we do not have data indicating which treatment was better for these patients.

### Conclusions

LD patients had higher in-hospital mortality compared with RD patients in a population with ACS patients; LD anatomy was an independent predictor for in-hospital mortality in the multivariate analysis in this Japanese, real-world, multicenter study. A single coronary supply, typical of patients with an LD anatomy, should be recognized as a high-risk feature.
